# Predictors of Employment for People With Mental Illness: Results of a Multicenter Randomized Trial on the Effectiveness of Placement Budgets for Supported Employment

**DOI:** 10.3389/fpsyt.2019.00518

**Published:** 2019-07-19

**Authors:** Wulf Rössler, Mariam Ujeyl, Wolfram Kawohl, Carlos Nordt, Antonio Lasalvia, Helene Haker, Michael P. Hengartner

**Affiliations:** ^1^Department of Psychiatry and Psychotherapy, Charité University Medicine Berlin, Berlin, Germany; ^2^Psychiatric Services Aargau, Windisch, Switzerland; ^3^Department of Psychiatry, Psychotherapy, and Psychosomatics, University of Zurich, Zurich, Switzerland; ^4^Department of Neuroscience, Biomedicine, and Movement Sciences, University of Verona, Verona, Italy; ^5^Translational Neuromodeling Unit, ETH Zürich, Zurich, Switzerland; ^6^Department of Applied Psychology, Zurich University of Applied Sciences, Zurich, Switzerland

**Keywords:** supported employment, vocational rehabilitation, placement budget, serious mental illness, predictors, prognosis

## Abstract

**Background:** Individual placement and support (IPS) has proven to be effective for vocational outcomes in people with mental illness. The original concept of IPS requires temporally unlimited provision of support. Using limited placement budgets and investigating factors that predict their effectiveness may inform decisions about resource allocation.

**Methods:** A range of patient characteristics were tested as predictors of employment outcomes in participants who attended six outpatient psychiatric clinics in Switzerland between June 2010 and May 2011. Overall, 116 patients with the full spectrum of psychiatric conditions were randomly assigned and started an IPS intervention, which was provided by three different placement budgets. Support lasted 2 years for those who found a job, and outcomes were repeatedly assessed over 3 years. The intervention ended for those who failed to find competitive employment by the time their placement budget had run out.

**Results:** Of the 15 variables tested, only Global Assessment of Functioning (GAF) and Clinical Global Impression (CGI) scores were predictors for obtaining work (for ≥1 day) and for maintaining it over a longer period (>3 months). Higher GAF and lower CGI scores increased the odds of obtaining employment and keeping it for at least 3 months. Functional role impairment, quality of life, self-esteem, or education level did not predict employment.

**Conclusion:** Our data suggest that, if time-restricted budgets are offered to a wide range of patients, such as those included in this study, better functioning and lower symptom severity at baseline are predictive of better employment outcomes (finding and maintaining work) on the first (competitive) labor market in Switzerland. It remains to be investigated whether this holds true under different environmental factors.

**Clinical Trial Registration:** ISRCTN, trial number: ISRCTN89670872.

## Introduction

Addressing mental ill health among the working-age population has become a key issue for labor market and social policies across Europe (1). In Switzerland, employment rates of people with mental disorders are relatively high compared to those in other European countries. But even under these beneficial circumstances, people with mental illness have an unemployment rate that is almost three times higher than the average population level (2).

Despite growing attention to the topic, knowledge about the relationship between mental illness, patient characteristics, and employment outcomes remains limited (2). Among existing policies and practices designed to help individuals with mental illness obtain and maintain employment, the individual placement and support (IPS) model of supported employment (SE) is the one best studied (3, 4). The EQOLISE trial has shown IPS to be more effective than vocational services in *obtaining* employment for patients with severe mental illness in a range of European countries, among those Switzerland (5). But the only variables in this trial that were able to predict which patients (irrespective of intervention) would do well and work for *at least one day* during the study period were previous work history (duration of unemployment) and proportion of social needs met (6). A follow-up of the Swiss cohort 2 years after the end of EQOLISE showed *retention* of any job to be very low in both intervention groups (7), thus precluding investigation of predictors for which patients benefit from vocational training over longer periods.

A previous study based on the sample receiving IPS presented herein found that patients who believed that their own actions influence outcomes (referred to as action-outcome expectancy) were more likely to work for at least 1 day during the study period. This expectancy was associated with higher motivation, female sex, higher income, and quality of life (QoL) (8). Recent reviews all found prior work experience, and some also found higher motivation or lower age predictive of higher levels of acquisition of competitive employment with SE (9–11). There is evidence that affective disorder (compared to other diagnoses) is a positive predictor for obtaining work in people with mental illness, but this has not been established for those enrolled in SE programs (12).

The original concept of IPS requires temporally unlimited provision of support and comes with considerable uncertainty as to who benefits most from the service. Using pre-defined limited budgets and investigating factors that predict its effectiveness may enhance allocative efficiency by informing decisions about resource allocation. The current multicenter trial provides data on patients in Switzerland who, under comparable conditions regarding environmental factors [such as local economy, labor laws, disability policies, etc. (13)], received a budget of 25, 40, or 55 h for job finding and placement within an otherwise standard IPS program. In the main analysis of this trial, we found that these different budgets did not meaningfully affect employment rates (14). In this paper, we aimed to explore which patient characteristics at baseline predict employment outcomes. Firstly, we investigated factors that predict relevant job acquisition (working for at least 1 day); secondly, factors that predict who retains his or her job over a time period of at least 3 months following successful placement assistance. As the probation period in Switzerland is usually 3 months, this threshold was used as the cutoff for defining long-term (>3 months) *versus* short-term (1 day–3 months) employment.

## Methods

### Participants and Procedure

**Figure 1:**
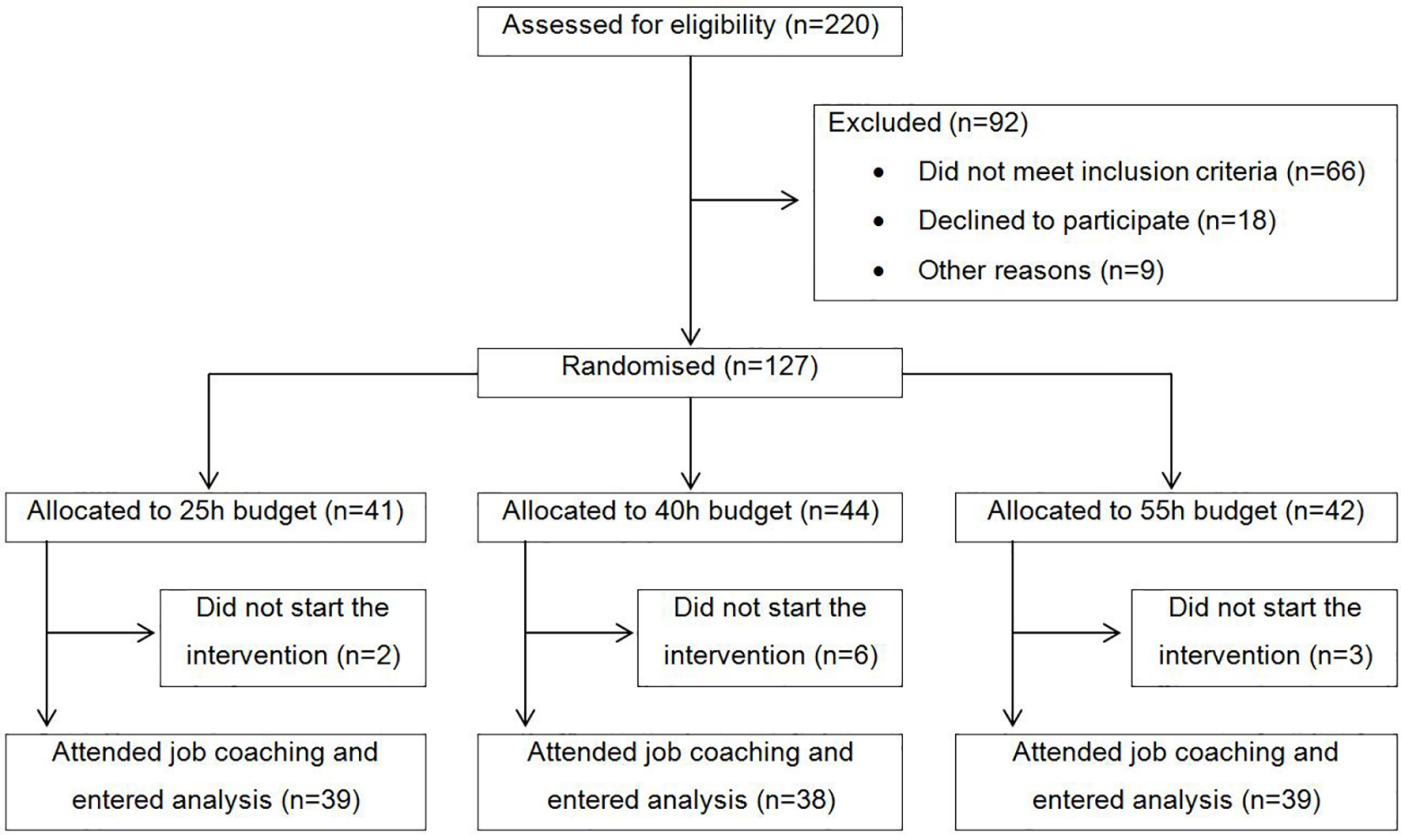
CONSORT participant flow chart.

Participants were recruited from six outpatient clinics in the canton of Zurich, Switzerland, between June 2010 and May 2011. Inclusion criteria were current treatment in one of the participating psychiatric outpatient clinics, at least 1 year of unemployment, no participation in a vocational integration program during the last 3 months, being of working age (i.e., 18–60 years), a desire to obtain competitive employment in the first (competitive) job market, being willing and able to give informed consent, and residing in the canton of Zurich. Exclusion criteria were severe organic illness und insufficient knowledge of the German language. All participants provided written informed consent. The CONSORT flow chart is shown in **Figure 1**. Altogether, 116 participants started the intervention and were included in our intent-to-treat analysis.

The specific intervention chosen for this trial is the IPS model (15) provided by different time-limited placement budgets, to which clients were randomly assigned (25, 40, or 55 h). The job coaches supported the clients for up to 2 years, or until the corresponding placement budget had run out for those who failed to find competitive employment. Patients were assisted for a maximum of 25, 40, or 55 h in job finding (i.e., work placement) and without time constraints in job maintenance (i.e., work support). That is, the time-restriction applied to assistance with the job search only (14). Job coaches were trained in the IPS model and had weekly meetings with supervision at the Supported Employment Department of the Psychiatric University Hospital of Zurich. In accordance with the IPS model, the job coaches assisted the participants in the following two tasks: first, time-restricted placement assistance (engagement, assessment, and finding a job that matches a client’s skills and interests), and second, unlimited support (help with maintaining competitive employment) for those who successfully started employment. Implementation fidelity was assessed every 3 months with the SE fidelity scale (14) and was uniformly high.

Altogether, seven assessments were conducted, that is, one baseline assessment (t0) and six follow-ups (t1–t6) every 6 months for a total observation period of 36 months. Retention was good, with n = 86 (74%) participants participating in the 24-month follow-up and n = 77 (66%) participating in the 36-month follow-up.

### Instruments and Measures

Irrespective of whether participants were still supported by their job coach, trained research assistants carried out assessments every 6 months over a total period of 3 years *via* computer-assisted face-to-face interviews. Employment status, duration of employment, and job description over the last 6 months were assessed *via* participants’ self-report. We applied two different outcome measures. The first outcome was employment for at least 1 day and was computed for the whole sample (n = 116) comprising the categories employed *vs*. unemployed. Based on the intent-to-treat principle, participants who dropped out of the trial before finding a job were counted as unemployed. The second outcome was computed only for people who obtained employment for at least 1 day (n = 67) and comprised short-term (<3 months) *vs*. long-term employment (≥3 months). We chose this dichotomization of employment duration because its distribution was bimodal. That is, participants either lost their job during the first 3 months (the usual probation period in Switzerland) or they remained employed for at least 1 year or longer. 3 months was therefore the appropriate cutoff to differentiate short-term from long-term employment.

Socio-demographic and clinical characteristics at baseline were derived from the German version of the Client Socio-Demographic and Service Receipt Inventory (CSSRI) (16) and from the Central Psychiatric Register of the Canton of Zurich (PSYREC), which includes the following information from clinical records: diagnosis according to ICD-10, Global Assessment of Functioning (GAF) score, and Clinical Global Impression (CGI). Severity of psychopathology was further assessed by self-report questionnaire using the Brief Symptom Inventory (BSI) (17).

Functional impairment was assessed by observer rating based on the short form of the International Classification of Functioning, Disability, and Health (Mini-ICF) (18), which is a valid and reliable instrument used to quantify disability, especially with regard to occupational functioning.

QoL was assessed with the German translation of the World Health Organization Quality of Life Bref (WHO QoL Bref) (19). This self-report questionnaire captures QoL based on four dimensions, specifically: 1) physical health (e.g., dependency on medical aids, energy and fatigue, mobility, work capacity), 2) psychological (e.g., negative and positive feelings, self-esteem, memory and concentration), 3) social relationships (e.g., personal relationships, social support, sexual activity), and 4) environment (e.g., financial resources, home environment, physical environment, safety and security). For the present analysis, we used only the total score. The WHO QoL has been shown to be a valid and reliable measure of QoL (19, 20).

Self-stigma was measured at baseline with the 29-item Internalized Stigma of Mental Illness scale (ISMI) (21). Its five subscales measure: 1) alienation, 2) stereotype endorsement, 3) perceived discrimination, 4) social withdrawal, and 5) stigma resistance. As reported in previous studies (21), the internal consistency of the stigma resistance subscale was low (Cronbach’s α = 0.49 in our study). Therefore, its five items were not included in the total score (α = 0.92).

Finally, self-esteem was assessed with the Rosenberg Self-Esteem Scale (RSE) (22). The RSE has shown good reliability and criterion validity (22, 23).

### Statistical Analysis

Associations between patient characteristics at baseline and job attainment over the 3-year observation period were analyzed with a series of binomial logistic regressions. Employment was entered as the dependent variable based on the two distinct definitions detailed above. We first regressed each predictor variable separately on employment. Statistically significant predictors at α = 0.05 were then included simultaneously in a multivariable regression model. The proportion of total variance explained (R^2^) was reported according to Nagelkerke’s R^2^ formula. All analyses were conducted with SPSS version 24 for Windows.

## Results

The descriptive statistics are shown in **Table 1**. Clinical characteristics varied greatly, with some patients being highly impaired and symptomatic, and others apparently having few symptoms and a high level of functioning. Only three patients (2.6%) were rated very low functioning (GAF < 30), whereas 10 (8.6%) were rated relatively high functioning (GAF > 70). With respect to the CGI, 3 patients (2.6%) were considered normal or borderline ill (CGI 1 or 2) and 25 (21.6%) were rated severely ill (CGI 6 or 7). Out of 116 participants, 49 (42.2%) did not find employment or dropped out within 3 years. Among the 67 (57.8%) participants who obtained employment, 21 (31.3%) had short-term employment only (1 day–3 months), and 46 (68.7%) obtained long-term employment (> 3 months). No participant worked for 1 day only, but for many people, employment ended shortly before 3 months, which is the usual probation period in Switzerland. According to a Χ^2^ test, employment (none *vs*. short *vs*. long) was not related to the different placement budgets (25 h *vs*. 40 h *vs*. 55 h), Χ^2^ = 3.48, df = 4, and p = 0.480. The proportion of participants who obtained competitive employment for at least 1 day was 64.1% in the 25-h group, 55.3% in the 40-h group, and 53.8% in the 55-h group. In total, 47.8% of all participants who found a job did so within the first 6 months and 80.4% did so within the first 12 months. No long-term employment was obtained after 24 months. The detailed findings for the effect of the different placement budgets on employment have been published elsewhere (14).

**Table 1:** Descriptive statistics at baseline (n = 116).

	N/range	%/Mean (SD)
Placement budget 25 h 40 h 55 h	N = 39 N = 38 N = 39	33.6% 32.8% 33.6%
Female sex (*vs*. male)	N = 59	50.9%
Partnership (*vs*. single)	N = 52	44.8%
Age	19-60	41.3 (10.4)
Years in education	7–22	11.4 (3.0)
Competitive job last year (*vs*. more than 1 year ago)	N = 20	17.2%
Total years unemployed	1–15	2.9 (2.6)
Net income in Swiss Francs per month*	0–10,600	2,567.8 (1,935.4)
Affective disorder (vs. other)^#^	N = 71	61.2%
Substance-use disorder (ICD-10 F1)	N = 12	10.3%
Schizophrenia and related disorders (ICD-10 F2)	N = 11	9.5%
Depressive disorder (ICD-10 F3)	N = 50	43.1%
Anxiety and stress-related disorders (ICD-10 F4)	N = 21	18.1%
Other diagnoses	N = 22	19.0%
CGI	1–7	4.9 (1.1)
BSI total score	1.1–4.0	2.0 (0.7)
GAF	20–85	56.1 (12.0)
ICF total score	0–33	13.4 (7.4)
QoL total score	1.0–4.5	3.0 (0.8)
ISMI total score	1.1–3.4	2.1 (0.5)
RSE total score	1.5–3.8	2.7 (0.5)

*1 Swiss Franc = 0.9 Euro.

^#^Affective disorder includes F3 and F4 diagnoses.

The 16 clinically relevant variables listed in **Table 1** were then regressed consecutively against obtaining a job for at least 1 day (yes *vs*. no). Significant associations were found for years in education (Χ^2^ = 4.24, df = 1, p = 0.040, R^2^ = 0.049), CGI score (Χ^2^ = 10.29, df = 1, p = 0.001, R^2^ = 0.114), BSI total score (Χ^2^ = 4.10, df = 1, p = 0.043, R^2^ = 0.047), GAF score (Χ^2^ = 10.89, df = 1, p = 0.001, R^2^ = 0.120), and ISMI total score (Χ^2^ = 4.26, df = 1, p = 0.039, R^2^ = 0.051). These variables were then entered simultaneously into a multivariable regression model. Because GAF and CGI were strongly correlated (Pearson r = −0.63, p < 0.001), we included only the GAF score to avoid multicollinearity issues. The model with four predictors was statistically significant and explained 17.2% of variance in obtaining a job for at least 1 day (Χ^2^ = 14.88, df = 4, p = 0.005, R^2^ = 0.172). The parameter estimates are shown in **Table 2**. When adjusted for each other, only the GAF score remained a significant predictor. A 1-point increase on the GAF scale increased the odds of obtaining employment by 5.1%. For a 10-point increase on the GAF scale, the odds ratio translates into an increase of 64.9%. When the CGI score was entered into the model instead of the GAF, it also emerged as the sole predictor.

**Table 2:** Multivariable predictor model for obtaining employment for at least 1 day (n = 116).

	OR	95% CI	*P*
Years in education	1.10	0.95; 1.28	0.211
BSI total score	0.78	0.35; 1.73	0.544
GAF	1.05	1.01; 1.09	0.012
ISMI total score	0.67	0.24; 1.87	0.441

We then tested whether the same predictors would relate to short (1 day–3 months) *versus* long-term employment (>3 months). When tested separately, significant associations were found for the CGI and GAF scores only. So, in contrast to the previous model, no associations were found for the BSI total score, ISMI total score, and years in education. The CGI explained 26.1% of variance in short- *versus* long-term employment0 (Χ^2^ = 13.75, df = 1, p < 0.001, R^2^ = 0.261) and the GAF accounted for 21.4% of total variance explained (Χ^2^ = 11.08, df = 1, p = 0.001, R^2^ = 0.214). The parameter estimates are shown in **Table 3**. Owing to the strong correlation between GAF and CGI, only bivariate associations are reported. One more point on the CGI scale reduced the odds of obtaining long-term employment by 69.6%, whereas one more point on the GAF scale increased the odds by 10.1%. For a 10-point increase on the GAF scale, this translates into an increase in the odds of 161.2%.

**Table 3:** Bivariate predictors of short (≤3 months) *versus* long-term (>3 months) employment (n = 67).

	OR	95% CI	*P*
CGI	0.30	0.14; 0.67	0.003
GAF	1.10	1.03; 1.18	0.004

## Discussion

The aim of this work was to examine predictors that relate to acquiring a job in the first (competitive) labor market for at least 1 day and to retaining this job for at least 3 months. The only factor that was independently related to both acquiring and retaining a job was the severity of illness as assessed with either CGI or GAF. That is, patients who experienced less distress and had better global functioning were more likely to find and keep a job. Factors such as self-esteem, internalized stigma, years in education, income, functional role impairment, duration of unemployment, or sex and age were not related to employment outcomes.

These results contrast with those from other relevant studies, which have usually included participants with comparable functioning (as assessed by GAF or CGI), but from a different diagnostic spectrum. In this study, we included participants from outpatient clinics, with the majority being diagnosed with either a mood or a neurotic, stress-related, or somatoform disorder (ICD-10 F3 or F4 diagnosis), and only a minority with a schizophrenia [ICD-10 F2 diagnosis; see Rössler et al. (25)]. Most other trials in SE have included only participants with severe mental illness, such as schizophrenia and bipolar disorder. For example, the vast majority of participants in the EQOLISE trial (5) had been diagnosed with an ICD-10 F2 disorder. In those patients, global functioning (GAF) and positive and negative syndrome scale (PANSS) were not predictive of entering competitive employment. It might be the case that the symptoms reflected by GAF and CGI scores act differently on employment prospects in patients with affective, neurotic, or stress-related disorders as compared to those with schizophrenia.

Another difference between this and other studies lies in the definition of outcomes. A strength of our study is that it examined not only employment for at least 1 day, as in most other studies in the field, but also job retaining, that is, employment for at least 3 months. As an additional strength of this study, we would like to emphasize its long-term follow-up with high retention rate. It is possible that variables predicting who will find a job for at least 1 day might not be as suitable for predicting who will benefit from IPS over a longer and more meaningful period. However, this study identified the same variables to predict who finds and who retains a job.

We also acknowledge the following major limitations. Owing to the small number of participants, the representativeness and generalizability of the sample are uncertain. In addition, this may have restricted the statistical power in detecting job predictors of weak effect size, especially as we have included a wide range of different diagnoses and, as such, a fairly heterogeneous sample. Finally, the present analysis was exploratory, and significant associations must therefore be interpreted with caution.

## Conclusion

We investigated predictors of the effectiveness of placement budgets for SE in a population of participants in different psychiatric outpatient clinics in Switzerland. Our data suggests that, if time-restricted budgets are offered to a wide range of patients, irrespective of diagnosis, better functioning and lower symptom severity at baseline are predictive of better employment outcomes (finding and maintaining work) in the first (competitive) labor market. Whether this relationship holds true under different environmental factors remains to be investigated.

## Author Contributions

CN, WK, and WR took responsibility for the study design. WR, MH, and MU took responsibility for the statistical analyses and drafted and revised the manuscript. CN and WK supervised the assessments, and substantially contributed to drafting and critical revision of the manuscript. HH took responsibility for special assessments and substantially contributed to drafting and critical revision of the manuscript. AL substantially contributed to drafting and critical revision of the manuscript.

## Ethics Statement

The trial was pre-registered in ISRCTN registry (trial number: ISRCTN89670872) and the study protocol was published freely available online. The study protocol was approved by the Zurich Cantonal Ethics Committee (CEC), reference number E-51/2009.

## Funding

The study is one of the six sub-projects of the Zurich Program for Sustainable Development of Mental Health Services (ZInEP), funded by a generous bequest from a foundation.

## Conflict of Interest Statement

The authors declare that the research was conducted in the absence of any commercial or financial relationships that could be construed as a potential conflict of interest.
